# Body Mass Index as an Example of a Negative Confounder: Evidence and Solutions

**DOI:** 10.3390/genes16050564

**Published:** 2025-05-10

**Authors:** Zhu Liduzi Jiesisibieke, C. Mary Schooling

**Affiliations:** 1 School of Public Health, Li Ka Shing Faculty of Medicine, The University of Hong Kong, Hong Kong, China; julie822@connect.hku.hk; 2 Graduate School of Public Health and Health Policy, City University of New York, New York, NY 10027, USA

**Keywords:** negative confounding, body mass index, physiological attributes, Mendelian randomization

## Abstract

**Background:** Adequate control for confounding is key to many observational study designs. Confounders are often identified based on subject matter knowledge from empirical investigations. Negative confounders, which typically generate type 2 error, i.e., false nulls, can be elusive. Such confounders can be identified comprehensively by using Mendelian randomization (MR) to search the wealth of publicly available data systematically. Here, to demonstrate the concept, we examined whether a common positive confounder, body mass index (BMI), is also a negative confounder of any common physiological exposures on health outcomes, overall and specifically by sex. **Methods:** We used an MR study, based on the largest overall and sex-specific genome-wide association studies of BMI (i.e., from the Genetic Investigation of ANthropometric Traits and the UK Biobank) and of relevant exposures likely affected by BMI, to assess, overall and sex-specifically, whether BMI is a negative confounder potentially obscuring effects of harmful physiological exposures. Inverse variance weighting was the main method. We assessed sex differences using a z-test. **Results:** BMI was a potential negative confounder for apolipoprotein B and total testosterone in men, and for both sexes regarding low-density lipoprotein cholesterol, choline, linoleic acid, polyunsaturated fatty acids, and cholesterol. **Conclusions:** Using BMI as an illustrative example, we demonstrate that negative confounding is an easily overlooked bias. Given negative confounding is not always obvious or known, using MR systematically to identify potential negative confounders in relevant studies may be helpful.

## 1. Introduction

Confounding, occurring when a factor influences both exposure and outcome, is a common challenge, especially in observational studies seeking to identify causal effects [1,2]. Confounding is a causal concept making identification solely from conventional observational studies difficult [3], particularly when relevant contextual knowledge is incomplete. Refutation of observational findings by randomized controlled trials has demonstrated the importance of key positive confounders, such as socioeconomic position, health status, and greater health consciousness, in generating false positives [4]. Conversely, negative confounding presents challenges in identification because it typically generates false nulls, which may not be further investigated, potentially creating type 2 error. Focus on finding and publishing statistically significant findings [5,6] may also lead to type 2 errors being overlooked, as apparently null associations may not appear worth investigating. In addition, negative confounding may be overlooked because a commonly cited example of negative confounding, involving nutrients from fish intake mitigating the effects of mercury toxicity on cognition [7], is not clearly confounding, given that the impact of mercury toxicity varies with the role of fish in the food chain. A recent study used a Mendelian randomization (MR) design to identify confounding [8]; however, it did not specifically address negative confounding.

Negative confounders are factors which bias the findings toward the null or inverse. A confounder that has directionally opposite associations with exposure and outcome will bias the association, when the exposure increases the outcome, towards the null or inverse. For example, BMI reducing low-density lipoprotein cholesterol (LDL-c) negatively confounds estimates of LDL-c on ischemic heart disease [9], obscuring the full harms of LDL-c.

To clarify and investigate the potential for negative confounding, as an exemplar, we investigated the role of one potential negative confounder, i.e., BMI, because BMI has previously been identified as a negative confounder [10,11], but has not been systematically investigated as such. Given that an MR study design is more robust to confounding than many other observational study designs, we used MR to systematically investigate whether BMI is a negative confounder of exposures, plausibly driven by BMI, i.e., physiological attributes. Given that body composition [12], metabolism [13], and health risks [14] may differ between men and women, BMI may confound differently in each sex. Therefore, we also considered negative confounding sex-specifically. For comparison and completeness, we also considered the role of BMI as a positive confounder.

## 2. Methods

### 2.1. Negative Confounding by BMI

**Figure 1:**
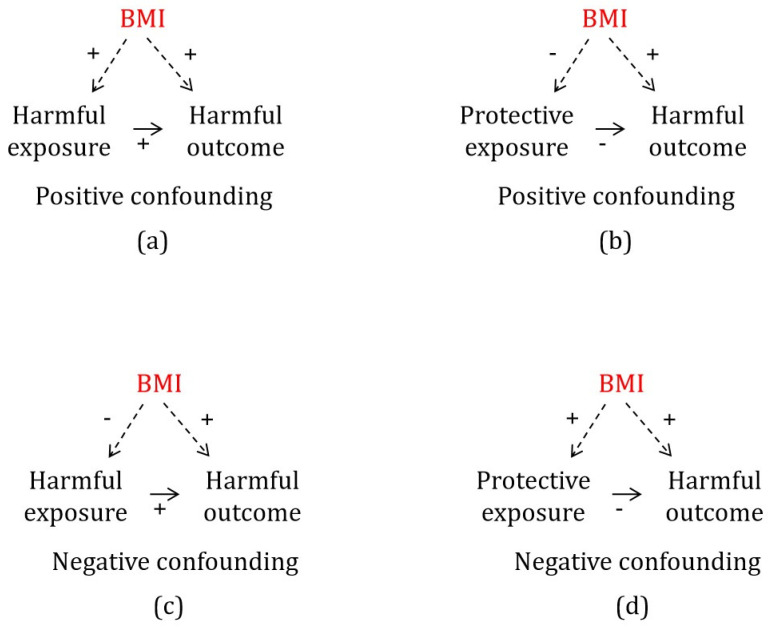
Directed acyclic graph of positive confounding (**a**,**b**) and negative confounding (**c**,**d**).

BMI is a positive confounder for a harmful outcome if it increases a harmful exposure or reduces a protective exposure; failure to adjust for a positive confounder biases the estimate away from the null (Figure 1a,b). In contrast, BMI is a negative confounder for a harmful outcome if it reduces a harmful exposure or increases a protective exposure; failure to adjust for a negative confounder biases the estimate towards the null or inverse (Figure 1c,d). Given that public health research tends to focus on means of improving population health, i.e., reducing harmful outcomes, we primarily considered negative confounders for harmful outcomes.

### 2.2. Study Design

To identify possible negative confounders of harmful outcomes by BMI, we systematically searched for potentially harmful exposures (such as apolipoprotein B (ApoB)) [15], which are reduced by BMI, or potentially beneficial exposures (such as ketones) [16,17], which are increased by BMI. We searched using MR because the use of genetic instruments largely obviates confounding [18]. The wealth of publicly available genome-wide association studies (GWASs) available for MR studies also enables a comprehensive search. To establish whether a factor is generally harmful or not, we assessed its effect on lifespan. Conversely, for completeness in identifying possible positive confounders of harmful outcomes by BMI, we searched systematically for potentially harmful exposures increased by BMI or potentially beneficial exposures that are reduced by BMI.

### 2.3. Assumptions of MR

MR uses genetic variants randomly assorted at conception as instrumental variables (IV). MR is based on three assumptions: relevance (genetic variants predict the exposure) [19], independence (genetic variants are free from confounding) [20], and exclusion restriction (genetic variants are independent of the outcome given the exposure and confounders) [21].

### 2.4. Data Sources

#### 2.4.1. Body Mass Index

This study utilized the largest sex-specific GWAS for BMI, including 194,174 women and 167,020 men (https://www.nealelab.is/uk-biobank, accessed on 26 May 2024), taken from the UK Biobank, a prospective cohort study of half a million adults [22,23]. The average age of participants was 57 years. Sex-specific phenotypes were adjusted for age, age^2^, and the first 20 principal components (Table 1), which represents a compromise between sufficient adjustment for population structure without also adjusting for linkage disequilibrium [24]. The UK Biobank is the largest source of exposures and outcomes, but when two-sample MR methods are applied in one study, they can be open to bias [25]. Therefore, we also replicated the analysis using sex-specific BMI from a non-UK Biobank study, i.e., Genetic Investigation of ANthropometric Traits (GIANT) (women: 171,977, men: 152,893) [26], which was adjusted for age, age^2^, and study-specific covariates (Table 1). We also considered sex-combined BMI, where we used the largest available GWAS (n: 681,275), also from GIANT [27], which was adjusted for age, age^2^, principal components, and study-specific covariates (Table 1).

#### 2.4.2. Inclusion and Exclusion Criteria for Exposures

We considered as exposures physiological factors potentially affected by BMI. We focused on phenotypes measured in blood or urine because they are most likely to be influenced physiologically by BMI, including metabolomics from the UK Biobank. We excluded behaviors and environmental attributes as unlikely directly driven by BMI, such as alcohol use, employment, or home environment.

**Figure 2:**
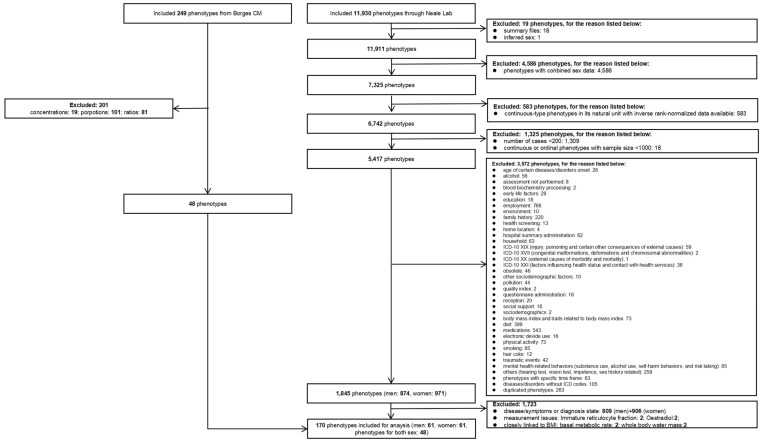
Phenotype screening process for inclusion.

We used sex-specific phenotypes in preference to sex-combined phenotypes. We only used rank-normalized continuous variables [28] to reduce the potential effects of outliers. We only used binary phenotypes with more than 200 cases and continuous phenotypes with sample size of more than 1000 [28]. International Classification of Disease (ICD) coded phenotypes without main ICD codes, duplicates, and factors, such as disease-related phenotypes unlikely to be exposures, were also excluded. We discarded duplicate phenotypes. We also discarded ratio measures because they are difficult to interpret [29]. Oestradiol and immature reticulocyte fractions were excluded due to known measurement issues [30,31]. We excluded basal metabolic rate because it is closely linked to BMI. Additionally, we excluded whole-body water mass, which is not a physiological attribute of primary concern. Detailed exclusion criteria are listed in Figure 2.

#### 2.4.3. Categorization of Exposures

Exposures considered were categorized as blood measurements, metabolomics, minerals, physical measurements, and vitamins.

#### 2.4.4. Selection of Genetic Instruments for BMI

We selected genetic instruments for BMI as independent SNPs (*r*^2^ < 0.001 within a 10,000 kb window), strongly (*p* < 5 × 10^−8^) associated with BMI from the largest available suitable GWAS. Perforce, we focused on Europeans, because of the availability of suitable GWAS.

#### 2.4.5. Statistical Analysis

The F-statistic, a measure of instrument strength, was calculated as the mean of *β*^2^ divided by the square of the standard error, where *β* is the coefficient for each genetic variant on exposure. An F-statistic larger than 10 represents adequate instrument strength [32]. Inverse variance weighting (IVW) was used to estimate the effects of BMI on the attributes considered. IVW relies on the Instrumental Strength Independent of Direct Effect (InSIDE) assumption, i.e., that the pleiotropic effects do not confound exposure on the outcome, and the average pleiotropic effect is zero [33]. The IVW estimate requires all genetic variants to be valid or to have balanced pleiotropy [34]. The weighted median and MR-Egger were used as sensitivity analyses. A weighted median estimate, very different from the IVW estimate, suggests that the IVW estimate is driven by outliers [35,36]. An MR-Egger estimate with a significant intercept indicates that the IVW estimate may be invalid [36,37]. We used I^2^_GX_ and MR-Egger to assess exclusion restriction [32]. We tested sex differences using a z-test [38]. We did not adjust for multiple comparisons because we are trying to identify false negatives rather than avoid false positives. The MR analysis utilized the R packages “TwoSampleMR” (v0.5.7), “Mendelian Randomization” (v0.9.0), and R version 4.3.0 (21 April 2023 ucrt) with the “ggplot2” package used for graphics. This study solely utilized publicly available data, eliminating the need for ethics approval.

## 3. Results

We used 134 and 147 SNPs for BMI in men and women from the UK Biobank. The F-statistics for BMI were above 10, indicating that the relevance assumption was satisfied.

### 3.1. Exposure Selection

Figure 2 shows the selection process for the exposures from the UK Biobank GWAS provided by Neale lab and Borges CM [39]. Of the 11,930 phenotypes provided by Neale lab, 19 phenotypes were excluded because they are not data. A total of 4586 phenotypes were excluded because they are sex-combined, and 583 were excluded because they are in natural units when standardized estimates are available. A total of 1325 phenotypes were excluded because they had fewer than 200 cases or a sample size under 1000. A total of 3572 phenotypes were excluded because they were irrelevant, such as age, ICD-10 classifications, environmental influences, and quality indicators. A total of 1723 phenotypes were excluded because they were disease/symptoms, diagnosis state, had measurement issues, or were closely linked to BMI. So, 122 phenotypes were selected as exposures from Neale Lab (29 blood measurements, 23 metabolomics, four minerals, four physical measurements, and one vitamin for men and women separately). Of the 249 phenotypes provided by Borges CM, 201 were excluded because they were ratios, proportions, or concentrations. We included 48 of these phenotypes for men and women together, as shown in Figure 2. So, in total, we had 122 sex-specific phenotypes as exposures from Neale Lab and 48 sex-combined phenotypes from Borges et al. For completeness, we also considered whether these exposures were open to positive confounding by BMI for a harmful outcome.

### 3.2. Negative Confounding by BMI for a Harmful Outcome

#### 3.2.1. Potentially Harmful Exposures Reduced by BMI

**Figure 3:**
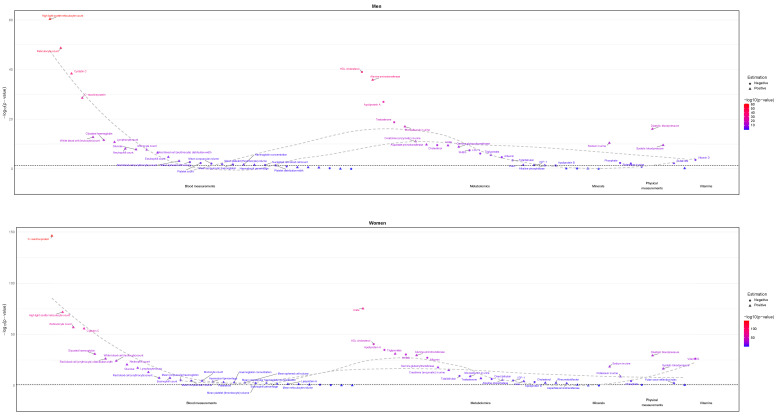
Sex-specific causal estimates for body mass index (women: 194,174; men: 167,020) on physiological attributes (women: 194,174; men: 167,020) from the UK Biobank.

Sex-specific BMI was inversely associated with several potentially harmful exposures in men, including ApoB [15], LDL-c [40], cholesterol [40,41], total testosterone [42], insulin-like growth factor 1 (IGF-1) [43], and platelet count [44] (Figure 3). Sex-specific BMI was inversely associated with several potentially harmful exposures in women, including cholesterol [40,41] and IGF-1 [43] (Figure 3). Estimates are shown in Supplementary Table S1.

**Figure 4:**
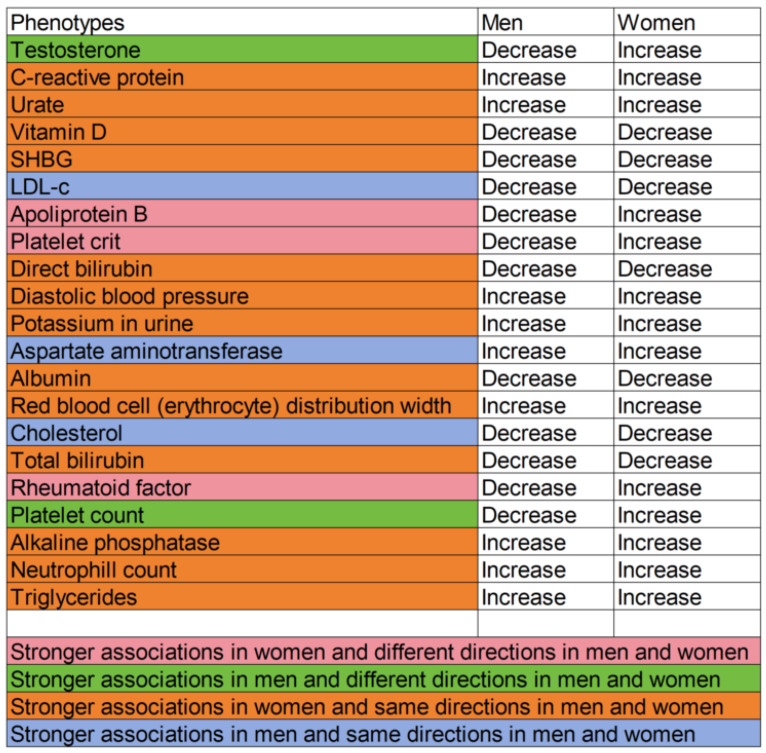
Significant sex differences in associations of body mass index (women: 194,174; men: 167,020) with physiological attributes (women: 194,174; men: 167,020).

Among these potentially harmful exposures reduced by BMI in men and women, which had a significant sex difference, estimates were generally stronger in women, albeit with notable exceptions, including LDL-c and cholesterol (Figure 4).

**Figure 5:**
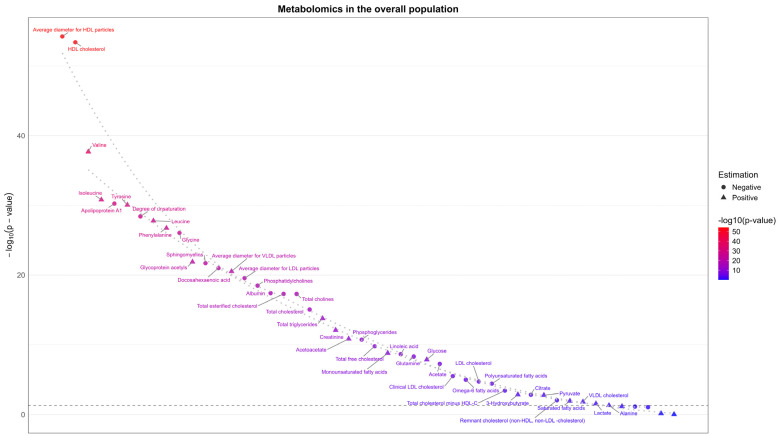
Causal estimates of body mass index (n: 681,275) from Genetic Investigation of ANthropometric Traits on phenotypes (n: 110,058~115,078) taken from a subsample of the UK Biobank.

Sex-combined BMI was inversely associated with several potentially harmful exposures in men and women together, i.e., total cholesterol [40,41], LDL-c [40], polyunsaturated fatty acids (PUFA) [39], glycine [45], linoleic acid [46], choline [47], and albumin [48] (Figure 5).

#### 3.2.2. Potentially Beneficial Exposures Increased by BMI

Sex-specific BMI was not positively associated with any potentially beneficial exposures in either men or women (Figure 3). Estimates are shown in Supplementary Table S1. Sex-combined BMI was positively associated with several potentially beneficial attributes, such as acetoacetate [16] and 3-Hydroxybutyrate [17] overall (Figure 5).

### 3.3. Positive Confounding by BMI for a Harmful Outcome

#### 3.3.1. Potentially Harmful Exposures Increased by BMI

As expected, sex-combined BMI was positively associated with many harmful or potentially harmful exposures, such as total triglycerides [49], glycoprotein acetyls [50], creatinine [51], monounsaturated fatty acids (MUFA) [52] in the overall population (Figure 5). BMI was also positively associated with ApoB [15], total testosterone [42], platelet crit [53] and rheumatoid factor [54] in women (Figure 3). Additionally, sex-combined BMI was positively associated with many potentially harmful attributes, such as valine, isoleucine, and leucine [45] (Figure 5).

#### 3.3.2. Potentially Beneficial Exposures Reduced by BMI

Sex-combined BMI was inversely associated with some potentially beneficial exposures, such as acetate [16] and high-density lipoprotein cholesterol (HDL-c) (with uncertainty about the benefits) [49] overall (Figure 5).

### 3.4. Sensitivity Analysis

For 4 of 122 physiological exposures considered (creatinine in men; pulse rate, nucleated red blood cell count, and LDL-c in women), the MR-Egger intercept was significant, while the IVW estimate was not (Supplementary Table S2). Results for BMI from the UK Biobank for physiological exposures using the weighted median were similar (Supplementary Table S3). IVW estimates for BMI from sex-combined GIANT on phenotypes from Borges et al. are shown in Supplementary Table S4. For none of the 48 phenotypes from Borges et al. considered as exposures was the MR-Egger intercept significant when the IVW estimate was not significant (Supplementary Table S5). Results for BMI from sex-combined GIANT on phenotypes from Borges et al. using the weighted median are shown in Supplementary Table S6. Given a few discrepancies between methods, possible horizontal pleiotropic effects could be incidental findings.

### 3.5. Replication

Using sex-specific BMI from GIANT yielded similar estimates to those derived from the UK Biobank; however, fewer SNPs resulted in wider 95% confidence intervals (CIs) (Supplementary Table S7). We used BMI from the UK Biobank due to its greater power, prioritizing the reduction in false negatives (i.e., potential type 2 error) over concern for false positives (i.e., type 1 error).

## 4. Discussion

As expected, BMI was potentially a positive confounder for several physiological attributes, such as HDL-c, which may have contributed to them being seen erroneously as protective. Importantly, we also found that BMI was potentially a negative confounder for associations with a harmful outcome for 13 of the 170 exposures considered. Exposures that could potentially be negatively confounded by BMI when considering harmful outcomes included ApoB, total testosterone, and platelet count in men; as well as cholesterol, IGF-1, LDL-c, PUFA, glycine, albumin, linoleic acid, choline, acetoacetate, and 3-Hydroxybutyrate in both men and women. As such, the harm of these exposures may not be fully appreciated, and of any other exposures similarly subject to negative confounding.

### 4.1. Comparison with Previous Studies

While many studies acknowledge pervasive positive confounding, such as by socioeconomic factors [55], the impact of negative confounding, has rarely been considered systematically. Positive confounding is well known to create type 1 error [56], which can be addressed by study design. However, negative confounding can mask true effects of the exposure of interest on health outcomes creating type 2 error. Specifically, the harms of ApoB and total testosterone in men, and of IGF-1, LDL-c, choline, cholesterol, linoleic acid, PUFA, glycine, and albumin in the overall population may not be fully identified in any study design open to confounding.

### 4.2. Implications for Observational Studies

Confounding is a crucial factor to consider when aiming to draw causal inferences from observational studies. Negative confounding is particularly hard to detect because it may generate type 2 error, where the exposure appears unrelated to the outcome, so it may be overlooked. Ignoring negative confounding can lead to false negatives, and exposures not being recognized as playing a role in disease when they do. Without external knowledge, negative confounders, such as BMI for some exposures, can be hard to detect. However, when external knowledge is lacking, MR can be used to assess whether potential confounders, particularly negative confounders, exist for a specific question, thereby reducing the risk of type 2 error and overlooking potential targets of intervention. Here, for demonstration purposes, we only consider one possible factor (BMI) as a possible negative confounder, so as to demonstrate how to identify negative confounding. However, to ensure all possible negative confounders are identified for a given research question, it would be necessary to use MR to search for the effects of all possible negative confounders on the association of the exposure with the outcome of interest.

### 4.3. Public Health Implications

Our study shows that negative confounding does occur. Our study also demonstrated how negative confounding can be identified without subject matter knowledge, by conducting an MR-PheWAS. Before adjusting for potential confounders in observational studies, it is advisable to employ MR as a tool to identify potential negative confounders, especially when experimental studies are not available. Such an approach may help facilitate the generation of valid results from observational studies.

### 4.4. Strengths and Limitations

Here, we demonstrated that MR could identify negative confounding, potentially reducing type 2 error. MR designs are generally robust to confounding. As such, MR is uniquely useful for identifying potentially overlooked negative confounders by conducting PheWASs to comprehensively identify all factors reducing a harmful exposure while increasing a harmful outcome, and all factors increasing a beneficial exposure while also increasing a harmful outcome. However, determining whether exposure and outcome meet these criteria still requires some subject matter knowledge, which can also be difficult to determine due to scarce evidence from RCTs or MR studies. Our specific approach is most practical for identifying confounders of physiological exposures and is limited by the genetic resources publicly available. Fortunately, large genetic studies for a wide variety of attributes across ethnicities are increasingly available. MR is also relevant to the general population rather than studies of patients because the underlying GWAS are usually obtained from population-based studies. Furthermore, MR is advantageous in that it captures lifelong implications of factors, such as BMI, which may differ from its short-term effects. However, MR is open to selection bias and competing risk, which can obscure the effects of harmful exposures, particularly at older ages [57]. Despite these limitations, MR provides another tool for identifying confounders in addition to subject matter knowledge or inference from observational data. In this study, we did not use a Bonferroni correction because we are trying to identify type 2 errors rather than to exclude false positives. Our study was also limited to Europeans, because of data availability; however, negative confounding should always be considered, although the sources may be contextually specific. Finally, accounting for negative confounding does not negate the role of positive confounding, selection bias, or effect modification.

## 5. Conclusions

Our study underscores the importance of recognizing and accounting for negative confounding in epidemiological research, as we illustrated for BMI as a potential negative confounder. To identify negative confounding, we advocate for future observational studies to acknowledge and systematically address this issue by using MR to identify negative confounders comprehensively and thereby facilitate a reduction of type 2 error. Prior observational studies, including some MR studies, may need to be reanalyzed to adjust comprehensively for all confounders (negative and positive) so as to obtain estimates with greater validity.

## Figures and Tables

**Table 1:** Data sources for body mass index used in this Mendelian Randomization study.

Trait	Data Source	Ancestry	Sex	Sample Size	Adjusted Covariates	R^2^	F Statistics in Univariable MR
Body mass index	UK Biobank (Neale lab)	European ancestry	Men	167,020	age, age^2^, and the first 20 principal components	4.1%	50.7
Body mass index	UK Biobank (Neale lab)	European ancestry	Women	194,174	age, age^2^, and the first 20 principal components	3.8%	49.8
Body mass index	GIANT [26]	European ancestry	Men	152,893	age, age^2^, and study-specific covariates	1.5%	62.8
Body mass index	GIANT [26]	European ancestry	Women	171,977	age, age^2^, and study-specific covariates	1.9%	65.7
Body mass index	GIANT [27] (includes approximately 64% from the UK Biobank)	European ancestry	Men and women	681,275	age, sex, and study-specific covariates	5.3%	72.8

## Data Availability

This study used data from the MR-base plat form (https://www.mrbase.org/), UK Biobank (http://www.nealelab.is/uk-biobank/), all accessed on 26 May 2024.

## Supplementary Materials

The following supporting information can be downloaded at: https://www.mdpi.com/article/10.3390/genes16050564/s1, Table S1. Causal estimates of body mass index (women: 194,174; men: 167,020) on physiological attributes (women: 194,174; men: 167,020) from the UK Biobank. Table S2. MR-Egger analysis of body mass index (women: 194,174; men: 167,020) on physiological attributes (women: 194,174; men: 167,020) from the UK Biobank. Table S3. Weighted median analysis of body mass index (women: 194,174; men: 167,020) on physiological attributes (women: 194,174; men: 167,020) from the UK Biobank. Table S4. Causal estimates of body mass index (n: 681,275) from Genetic Investigation of ANthropometric Traits on phenotypes from Borges et al. in both men and women (n: 110,058~115,078) using inverse variance weighting. Table S5. MR-Egger analysis of body mass index (n: 681,275) from Genetic Investigation of ANthropometric Traits on phenotypes from Borges et al. in both men and women (n: 110,058~115,078). Table S6. Weighted median analysis of body mass index (n: 681,275) from Genetic Investigation of ANthropometric Traits on phenotypes from Borges et al. in both men and women (n: 110,058~115,078). Table S7. Causal estimates of body mass index (women: 171,977; men n = 152,893) from Genetic Investigation of ANthropometric Traits on physiological traits (UK Biobank: women n = 194,174; men: 167,020).

## Abbreviations

ApoA1: apolipoprotein A; BMI: body mass index; CAD: cardiovascular diseases; GIANT: Genetic Investigation of ANthropometric Traits; GWAS: genome-wide association study; HDL-c: high-density lipoprotein cholesterol; IV: instrumental variables; IVW: Inverse variance weighting; LDL-c: low-density lipoprotein-cholesterol; MR: Mendelian Randomization; MUFA: monounsaturated fatty acids; PUFA: polyunsaturated fatty acids; RCT: randomized controlled trials; SD: standard deviation; SHBG: sex hormone-binding globulin.
